# Untrimmed ITS2 metabarcode sequences cause artificially reduced abundances of specific fungal taxa

**DOI:** 10.1128/aem.01537-24

**Published:** 2024-12-26

**Authors:** Kathleen E. Kyle, Jonathan L. Klassen

**Affiliations:** 1 Department of Molecular and Cell Biology, University of Connecticut 124501 , Storrs, Connecticut, USA; 2 Institute for Systems Genomics, University of Connecticut 703792 , Storrs, Connecticut, USA; Royal Botanic Gardens, Surrey, United Kingdom

**Keywords:** metabarcoding, ITS2, trimming, *Trichoderma*, fungi, bioinformatics

## Abstract

Advances in DNA metabarcoding have greatly expanded our knowledge of microbial communities in recent years. Pipelines and parameters have been tested extensively for bacterial metabarcoding using the 16S rRNA gene and best practices are largely established. For fungal metabarcoding using the internal transcribed spacer (ITS) gene, however, only a few studies have considered how such pipelines and parameters can affect community prediction. Here, we report a novel bias uncovered during ITS region 2 (ITS2) sequencing of *Trichoderma*-infected ant fungus gardens and confirmed this bias using mock communities. Abnormally low forward read quality caused *Trichoderma* ITS2 reads to be computationally filtered before and during read pair merging, thus almost entirely eliminating *Trichoderma* amplicon sequence variants from the resulting fungal community profiles. Sliding window quality trimming before filtering allowed most of these reads to pass filtering and merge successfully, producing community profiles that now correlated with visual signs of *Trichoderma* infection and matched the composition of the mock communities. Applying such sliding window trimming to a previously generated environmental ITS2 data set increased the detected fungal diversity and again overcame read quality biases against *Trichoderma* to detect it in nearly every sample instead and often at high relative abundances. This analysis additionally identified a similar, but distinct, bias against a second fungal genus *Meyerozyma*. The prevalence of such quality biases against other fungal ITS sequences is unknown but may be widespread. We, therefore, advocate for the routine use of sliding window quality trimming as a best practice in ITS2 metabarcoding analysis.

**IMPORTANCE:**

Metabarcode sequencing produces DNA abundance profiles that are presumed to reflect the actual microbial composition of their corresponding input samples. However, this assumption is not always tested, and taxon-specific biases are often not apparent, especially for low-abundance taxa in complex communities. Here, we identified internal transcribed spacer region 2 (ITS2) read quality aberrations that caused dramatic reductions in the relative abundances of specific taxa in multiple data sets characterizing ant fungus gardens. Such taxon-specific biases in read quality may be widespread in other environments and for other fungal taxa, thereby causing incorrect descriptions of these mycobiomes.

## INTRODUCTION

Fungal classification is notoriously difficult (1, 2), which may partly explain why fungi remain understudied compared to bacteria (3–6) despite their global importance in terrestrial and plant-associated ecosystems, including agriculture (7–9). Community amplicon sequencing, or “metabarcoding,” has been widely used to characterize bacterial communities using the 16S rRNA gene, which contains common bacterial barcode regions (10–12). Metabarcoding was later adapted to fungal communities, especially using the internal transcribed spacer (ITS) region of the eukaryotic rRNA gene cluster (13). Despite its later adoption, ITS metabarcoding is now one of the most widely used techniques for characterizing microfungal communities (14–16).

There are many biases associated with DNA metabarcoding (17–19). From the method of DNA extraction to the many challenges of PCR, biases in library generation and sequencing are well-documented (14, 17, 20–24). Historically, most studies of computational biases focused on the algorithms used to bin sequences into operational taxonomic units or exact/amplicon sequence variants (E/ASVs) (25–27), and those used for taxonomic classification (28, 29). Some recent studies have also considered biases associated with the upstream data manipulation steps (30–34). These upstream steps are broadly referred to as “preprocessing” because they are performed before the more computationally intensive binning and classification steps, and include checking the raw sequencing data for quality concerns and, if overlapping paired-end reads are available, merging reads into a consensus sequence.

Preprocessing standards have largely been developed for and adopted from bacterial metabarcoding (35–39). However, fungal ITS sequences pose extra challenges, particularly due to their length heterogeneity. For example, the widely used v4 region of the bacterial 16S rRNA gene (hereafter “16S v4”) is consistently ~250 bp long, but the comparable fungal rRNA internal transcribed spacer region 2 (hereafter “ITS2”) can range from ~50 to 800 bp long (40, 41). Thus, Illumina paired-end reads used to sequence 16S v4 metabarcodes overlap significantly with each other such that once merged, nearly every base is sequenced twice for improved basecall accuracy. When using a similar sequencing approach for ITS2 metabarcoding, sequences ~250 bp long can be merged and, therefore, sequenced twice. However, if the ITS2 gene is <250 bp, then the sequencing reads will contain non-biological bases when sequencing progresses into the 3′ adapter/primer region (“primer readthrough”; 42) and possibly beyond. In contrast, if the ITS2 sequences are too long, then the paired reads will share little or no overlap, reducing the proportion of bases sequenced twice and making merging difficult or impossible (43). This has led some researchers to question the value of using paired-end sequencing of ITS2 barcodes (44–46).

Successful read merging also depends on read quality. During preprocessing, low-quality bases are typically “trimmed” from the beginning and end of each read, and primer readthrough is sometimes “clipped.” Entire reads that still do not meet specified quality thresholds are then “filtered” out from the data set. Because the length of the 16S v4 metabarcode is homogeneous, many pipelines remove the same number of bases from the ends of all reads to remove bases at the 3′ read end that have the lowest quality scores. This produces higher-quality reads of a fixed length (36, 37, 47–51). In contrast, truncation of ITS2 reads to a fixed length is inappropriate because they have more variable lengths. Thus, ITS pipelines often omit this truncation step and proceed with untrimmed reads (although some do clip primer readthrough; 42, 46, 52, 53).

Using untrimmed metabarcoding reads has several downsides. At every step, compute times are increased by the unnecessary presence of low-quality bases. Additionally, filtering that only considers the average quality of entire reads will remove many untrimmed reads that typically have lower-quality bases at their 3′ ends, notwithstanding many high-quality bases at their 5′ ends. Untrimmed reads that pass filtering will then be more challenging to merge properly, if at all, due to the inclusion of these low-quality bases and adapter and primer sequences if they were not clipped. Finally, these merged sequences will inherit the lower quality of the untrimmed reads, potentially leading to inaccurate taxonomic binning and classification. For ITS barcodes specifically, filtering may be especially inaccurate if pipeline parameters are not changed from defaults chosen for 16S data sets. More fundamentally, truncating reads to a uniform length for the purpose of removing the lower-quality ends of reads assumes that read quality varies similarly for all reads in a data set.

Here, we report an underappreciated taxon-specific filtering bias during ITS2 metabarcoding (but see 54). In our previous research (55), we infected ant fungus gardens with *Trichoderma* (Ascomycota: Sordariomycetes: Hypocreales: *Hypocreaceae*), leading *to Trichoderma* growth that was visually apparent, yet there were no *Trichoderma* reads after ITS2 metabarcoding. We determined that *Trichoderma* reads in these samples uniquely failed to pass the filtering and read merging steps in our analysis pipeline, removing nearly all *Trichoderma* sequences from the final output. Using sliding window quality trimming before filtering remedied this bias against *Trichoderma* in both defined mock communities and our experimental infection data set. Furthermore, we detected the same bias against *Trichoderma* in an ITS2 metabarcoding data set from environmental samples, as well as a similar bias against *Meyerozyma* (Ascomycota: Saccharomycetes: Saccharomycetales: *Saccharomycetaceae*) that was also remedied by sliding window quality trimming. This study demonstrates how a taxon-specific bias due to an unusual reduction in quality at the 3′ end of ITS2 metabarcoding reads was not accommodated by typical filtering parameters, which led to erroneous taxonomic profiles and, thus, erroneous biological conclusions.

## RESULTS

**Fig 1:**
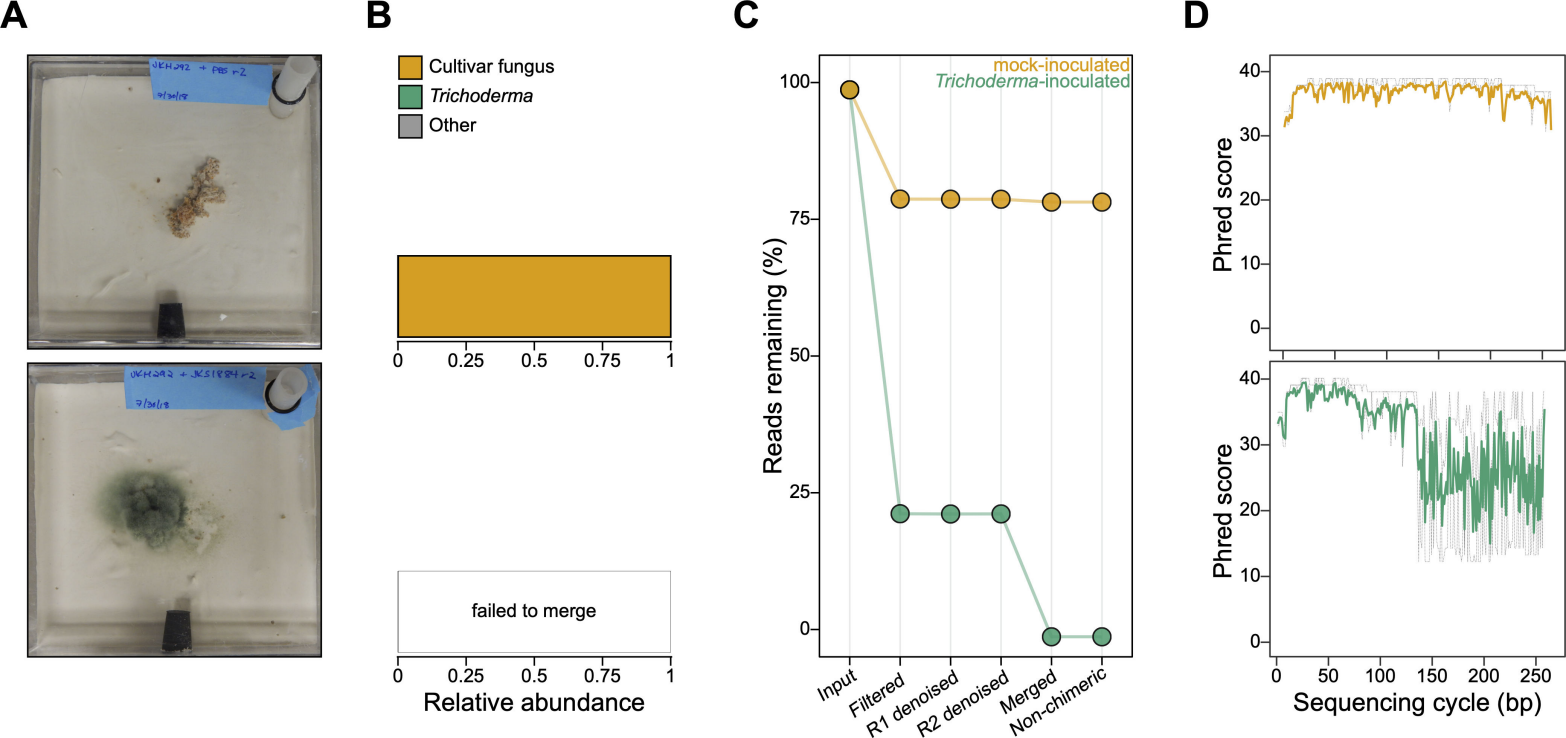
*Trichoderma* ITS2 reads are completely removed by filtering or failure to merge during metabarcode sequencing analysis and have abnormal forward read quality. (**A**) Representative images of a healthy fungus garden 4 days after inoculation with sterile PBS (“mock-inoculated,” top) and an infected fungus garden 4 days after inoculation with *Trichoderma* spores in PBS (“*Trichoderma*-inoculated,” bottom). (**B**) Relative abundances of fungal ASVs for the mock-inoculated (top) and *Trichoderma*-inoculated (bottom) fungus gardens pictured in (**A**). (**C**) Percent of reads remaining after each step of the metabarcoding analysis pipeline for the samples pictured in (A). Forward and reverse reads are abbreviated as R1 and R2, respectively. (**D**) Quality plots for forward ITS2 reads from the samples pictured in (**A**). Reads from the mock-inoculated (top) and *Trichoderma*-inoculated (bottom) samples are plotted in orange and green, respectively. The solid line shows the median basecall quality score (Phred) at each base position and dotted gray lines show the basecall quality quartiles. See Fig. S1 for results from the full data set.

During our previous work infecting fungus gardens cultivated by *Trachymyrmex septentrionalis* ants with the fungal pathogen *Trichoderma* (55), we visually observed *Trichoderma* growth on infected fungus gardens and not on control fungus gardens inoculated only with buffer (Fig. 1A). We were, therefore, surprised when our ITS2 community profiles for the infected fungus gardens contained very few *Trichoderma* ASVs (Fig. 1B; Fig. S1A), despite their containing the expected ASVs from the ant’s cultivar fungus (the main constituent of ant fungus gardens). Compared to the mock-inoculated controls, there were also many fewer reads in infected samples after filtering, with nearly all reads removed after paired read merging (Fig. 1C; Fig. S1B). The infected samples also had distinctively abnormal read quality profiles. At ~125 bp, forward read quality suddenly became highly variable and the median decreased sharply from a Phred score of ~35 to a score of ~25 that then persisted to the ends of these 250 bp reads (Fig. 1D; Fig. S1C). The quality of the reverse reads was poorer than the quality of the forward reads in every sample, but the quality of reverse reads from infected samples was noticeably poorer than that of reverse reads from the uninfected samples (Fig. S1C). These data suggested an unexpected and dramatic bias against ITS2 reads from *Trichoderma* in this analysis.

**Fig 2:**
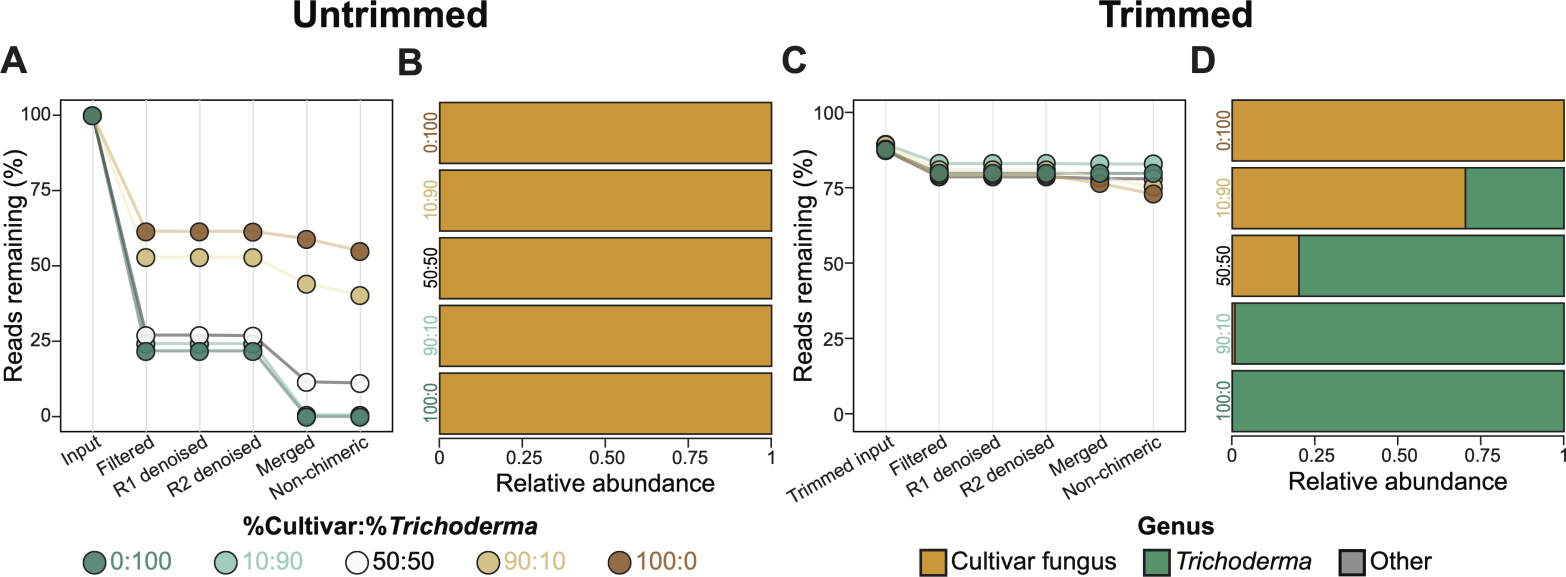
Mock communities replicate the bias against *Trichoderma* ITS2 metabarcodes and demonstrate that sliding window trimming can mitigate it. (**A**) Percent of untrimmed reads remaining at each step of the metabarcoding analysis pipeline for mock communities constructed using different proportions of cultivar fungus and *Trichoderma* DNA. (**B**) ASV relative abundances in each mock community using untrimmed reads. (**C**) Percent of trimmed reads remaining at each step of the metabarcoding analysis pipeline for the same mock communities as in (**A**). (**D**) ASV relative abundances in each mock community using trimmed reads. Forward and reverse reads are abbreviated as R1 and R2, respectively.

To reproduce this *Trichoderma*-specific bias more quantitatively, we sequenced mock communities created using defined proportions of DNA from pure cultures of *Trichoderma* and the ant cultivar fungus. As expected, the reduction in quality midway through the forward reads and the number of reads discarded during filtering and merging both increased alongside the proportion of *Trichoderma* DNA in the mock communities (Fig. 2A; Fig. S2). The input 100% *Trichoderma* sample had 112,972 read pairs, of which 24,632 passed filtering and only 20 merged successfully. For comparison, the input 100% cultivar fungus sample had 103,784 read pairs, of which 63,818 passed filtering and 61,220 merged successfully. No *Trichoderma* ASVs were detected in any of the mock communities, including that containing 100% *Trichoderma* DNA (Fig. 2B). Somewhat surprisingly, we detected cultivar fungus ASVs in the 100% *Trichoderma* mock community, but at very low levels likely originating from cross-contamination during community generation or sequencing.

We hypothesized that the sudden quality drop in the middle of the forward *Trichoderma* reads caused most of them to fall below the default quality thresholds and, thus, be filtered out or fail to merge, causing low final *Trichoderma* read counts and the absence of *Trichoderma* ASVs. To test this, we first quality trimmed the 3′ end of all reads in the mock community samples using the Trimmomatic’s sliding window trimmer (56) and then analyzed them as before. Sliding window quality trimming reduced the drop in read quality midway through the forward reads (Fig. S3), and most trimmed reads successfully passed the read filtering and merging steps of the analysis pipeline (Fig. 2C). The input 100% *Trichoderma* sample had 99,928 trimmed read pairs, of which 93,695 passed filtering and 93,673 merged successfully. The input 100% cultivar fungus sample had 92,100 trimmed read pairs, of which 85,456 passed filtering and 82,922 merged successfully. *Trichoderma* ASVs were now detected in all mock communities except for that containing 100% cultivar fungus DNA and their relative abundances correlated with the expected cultivar fungus:*Trichoderma* ratios of the input DNA (Fig. 2D), albeit with some overrepresentation of *Trichoderma*, likely because the communities were constructed using mass concentrations of genomic DNA instead of molar concentrations of the ITS2 region specifically. Such proportions based on genomic DNA mass concentration will be affected by different genome sizes and ITS2 copy numbers in *Trichoderma* versus those of the cultivar fungus. Overall, sliding window trimming generated metabarcoding community profiles that were much closer to the expected values compared to the profiles generated without such trimming (Fig. 2).

We next applied sliding window trimming to our initial *Trichoderma*-infection data set. Now, *Trichoderma* ASVs were detected at relative abundances consistent with the visual appearance of these samples (Fig. S4A), and the number of trimmed reads that were retained after filtering and merging (Fig. S4B) and the average quality of the trimmed forward reads (Fig. S4C) both increased for all *Trichoderma*-infected samples. Thus, sliding window trimming successfully mitigated bias against *Trichoderma* in these experimental samples, as it did for the mock communities.

**Fig 3:**
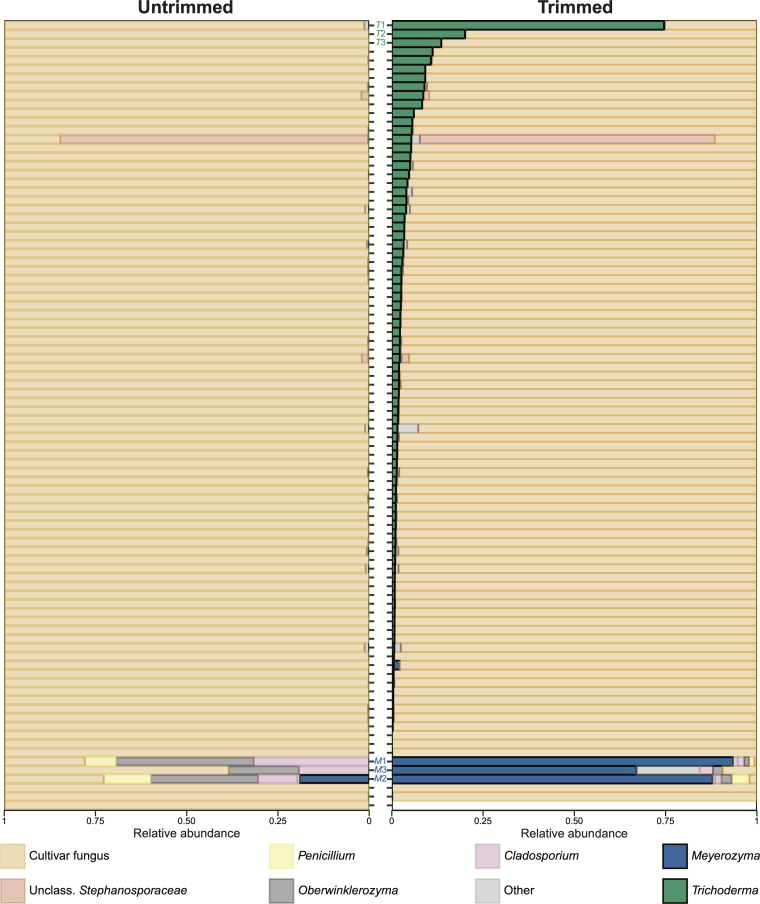
Using trimmed reads revealed a greater diversity of non-cultivar fungi in environmental fungus gardens compared to using untrimmed reads. ASV relative abundances in environmental fungus gardens using untrimmed (left) and sliding window-trimmed (right) reads. Each row represents an individual fungus garden, which is ordered by the relative abundance of *Trichoderma* after trimming. The samples with the three highest relative abundances of *Trichoderma* and *Meyerozyma* are labeled T1–T3 and M1–M3, respectively. The taxon labeled “Other” includes all ASVs that were <1% abundant in all samples. See Fig. S5 and Table S1 for the absolute abundances of fungal reads in this data set.

**Fig 4:**
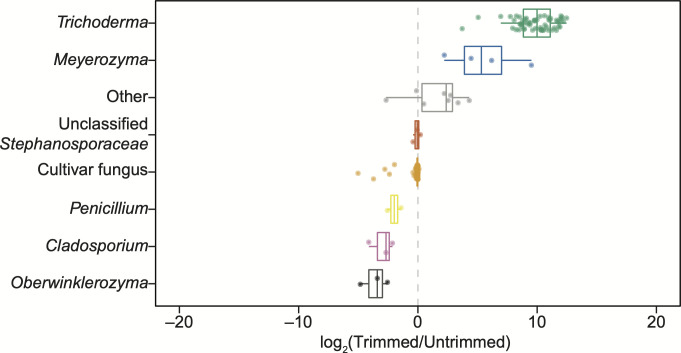
Trimming particularly increases the relative abundances of *Trichoderma* and *Meyerozyma*. The relative abundances of genera in the environmental samples (Fig. 3) were compared using their log_2_-transformed fold-change in trimmed versus untrimmed data sets, with each dot comparing the relative abundance of a genus in a single fungus garden sample. The center line and outer edges of the boxplots show the median and quartiles of the fold-change in relative abundances for each genus, respectively. Whiskers extend to the highest and lowest data points no further than 1.5× the interquartile range. Genera were excluded if they were <1% abundant in both untrimmed and trimmed data sets. Taxon labels and colors match those in Fig. 3 and genera are ordered by decreasing median log_2_-transformed fold-change.

Finally, we tested the effect of sliding window trimming on an environmental ITS2 data set that we previously generated from 98 freshly excavated ant fungus garden samples (55). Without trimming, very little taxonomic diversity appeared in this environmental data set (Fig. 3, left), with 86/90 samples having ≥98% cultivar reads, 3 samples containing <30% cultivar reads, and 1 sample having 62% cultivar reads. In contrast, considerably, greater taxonomic diversity was apparent after sliding window trimming, particularly for *Trichoderma,* which occurred in nearly all trimmed samples (81 out of 90) at often high relative abundances (Fig. 3, right). Similarly, trimming increased the relative abundance of the yeast genus *Meyerozyma* in four samples. One sample had 19% *Meyerozyma* before trimming which increased to 88% after trimming, and three samples went from 0% to 1.5%, 67%, or 93% *Meyerozyma*. Log2-transformed fold changes of trimmed versus untrimmed relative abundances of the genera in these environmental samples confirmed that *Trichoderma* and *Meyerozyma* relative abundances increased following sliding window trimming (Fig. 4). In this analysis, the genera whose relative abundances decreased after read trimming (cultivar fungus, *Penicillium*, *Cladosporium*, and *Oberwinklerozyma*) occurred in the same samples that had increased abundances of *Trichoderma*, *Meyerozyma*, and/or “other” fungi due to the relative nature of the metabarcoding data (i.e., because the samples must still sum to one, if the absolute abundance of *Trichoderma* or *Meyerozyma* increases after trimming, then the relative abundances of other taxa must decrease).

**Fig 5:**
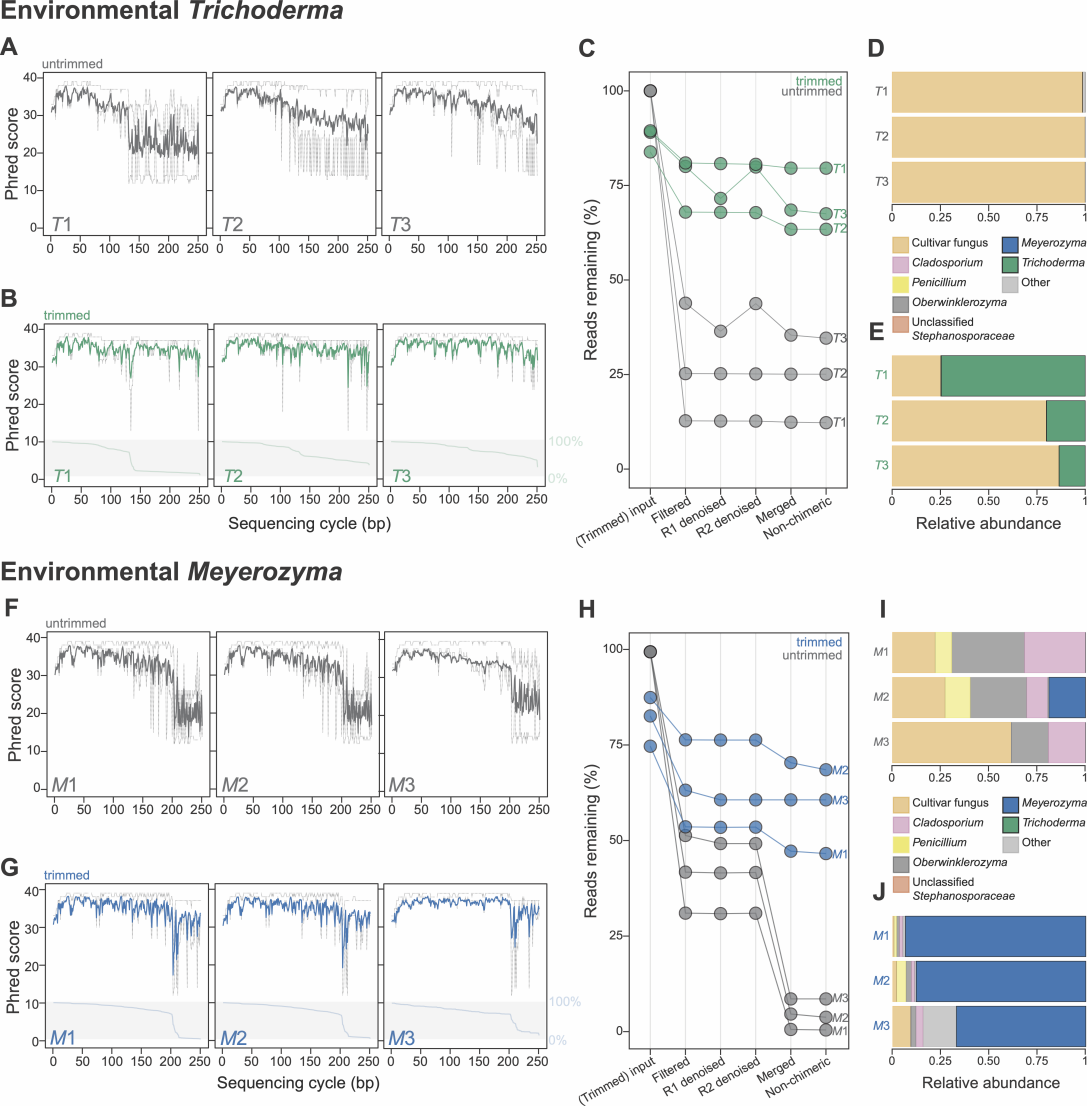
Trimming increases forward read quality and the number of reads that pass filtering and merge successfully for the environmental samples with the highest relative abundances of *Trichoderma* (***T*1–*T*3**) and *Meyerozyma* (***M*1–*M*3**). (A) and (B) Forward read quality plots for untrimmed (**A**) and trimmed (**B**) samples *T*1–*T*3. (**C**) Percent of reads remaining after each analysis pipeline step for trimmed and untrimmed samples *T*1–*T*3. (D) and (E) Relative abundances of fungal ASVs in untrimmed (**D**) and trimmed (**E**) samples *T*1–*T*3. (F) and (G) Forward read quality plots for untrimmed (**F**) and trimmed (**G**) samples *M*1–*M*3. (**H**) Percent of reads remaining after each analysis pipeline step for trimmed and untrimmed samples *M*1–*M*3. (I) and (J) Relative abundances of fungal ASVs in untrimmed (**I**) and trimmed (**J**) samples *M*1–*M*3. In panels (A), (B), (C), (F), (G), and (H), untrimmed data are colored gray and trimmed data are colored green (samples *T*1–*T*3) or blue (samples *M*1–*M*3). In panels (A), (B), (F), and (G), the median quality and the quality quartiles per base are plotted as solid and dotted lines, respectively. The light green or blue lines at the bottom of panels (B) and (G), respectively, show the percentage of reads trimmed to that sequence length or longer. Forward and reverse reads are abbreviated as R1 and R2, respectively.

Closer inspection of the environmental samples with the highest abundances of either *Trichoderma* (*T*1*–T*3) or *Meyerozyma* (*M*1*–M*3) mirrored the changes we observed in our infection and mock community experiments following sliding window trimming (Fig. 5). Left untrimmed, these environmental samples all had distinctive drops in the quality of the forward reads (Fig. 5A and F) and high numbers of reads discarded during filtering and merging (Fig. 5C and H; Table S1), making both taxa underreported in the relative abundance plots (Fig. 5D and I). Notably, the quality plots for samples containing *Meyerozyma* were similar to, but distinct from, those containing *Trichoderma,* with the sudden quality drop of the forward reads occurring at ~200 bp for *Meyerozyma* (Fig. 5F) compared to at ~125 bp for *Trichoderma* (Fig. 1C and 5A; Fig. S1C). Sliding window trimming mitigated both taxon-specific biases by improving the quality of the forward reads (Fig. 5B and G) and increasing read retention during read filtering and merging (Fig. 5C and H; Table S1). The resulting relative abundances of both taxa were much higher (Fig. 5E and J) compared to the untrimmed samples. Given our detection of such biases in samples that contained only limited taxonomic diversity, we speculate that similar metabarcoding read quality biases may exist for many other fungal taxa.

## DISCUSSION

This study demonstrates that data preprocessing, particularly sliding window quality trimming, can significantly affect fungal metabarcoding data quality and analytical output. The taxon-specific ITS2 read quality biases that we identified (Fig. 1D, Fig. 5A and F; Fig. S1 and S2) required these reads to be trimmed without applying a fixed truncation length to the entire data set to avoid taxon-specific read loss during downstream filtering and merging (Fig. 1C and 2A, Fig. 5C and H; Fig. S1B) and ultimately the underrepresentation or the absence of these taxa in the resulting ITS2 community profiles (Fig. 1B, 2B, and 3, Fig. 5D and I; Fig. S1A). In fact, sliding window quality trimming improved read retention after filtering and merging for all taxa in our data sets (Fig. 2C; Fig. S4B and S5) and improved classification of the cultivar fungus (Fig. S6), demonstrating its general benefit compared to fixed length truncation. So long as a metabarcoding pipeline can tolerate reads with variable lengths, sliding window quality trimming should apply to metabarcoding using any barcode. We did not detect “ASV splitting” following trimming (Fig. S6), which has been thought to arise from variable metabarcode read lengths (57, 58). Concatenating paired reads, alongside or instead of merging, may also improve taxonomic classification (30, 57), particularly for long amplicons that do not overlap enough for merging (34). This likely depends on gap size, however, because very large gaps can disrupt high-scoring alignments. That most reads in our study successfully merged after trimming (Fig. 2C, Fig. 5C and H; Fig. S4B; Table S1) suggests that the abnormally low forward read quality we observed is instead likely due to sequencing progressing beyond the end of short ITS2 template DNA molecules.

Our results additionally emphasize the importance of sequencing mock community controls alongside experimental samples (Fig. 2). Mock community controls are a gold standard tool to identify taxon-specific biases and validate computational pipelines (59). Ideally, these communities should contain every taxon present in the experimental communities being analyzed. Paradoxically, this requires prior knowledge of experimental community composition that is often unavailable, especially for understudied communities. Alternatively, constructing mock communities that include all currently known taxa is as impractical as it is impossible. Custom mock communities offer some promise (30, 60, 61), but these still require *a priori* knowledge of community composition, are technically challenging to create, are not standardized across research groups, and are currently rare for fungi (ATCC MSA-1010 and MSA-2010 from American Type Cultivar Collection, Manassas, VA, USA; 40, 62). Even if not comprehensive, mock community controls are useful to detect biases against the taxa that they do contain and, thus, should be used routinely.

When representative mock community controls do not exist, mindful data analysis is imperative. Researchers analyzing metabarcoding data should perform sufficient quality control during all steps of a computational analysis and especially appreciate that the default parameters are often set using well-characterized bacterial communities that may not apply to studies of other communities, particularly those targeting fungi (see [54] for an example of parameter optimization). These quality checks should include, but are not limited to, evaluating each sample for unusual patterns of read quality, tracking how many reads are removed from samples at each pipeline step, and comparing final community composition metrics between data sets processed using different parameters (e.g., trimmed versus untrimmed). Results also should be examined carefully with respect to known sequencing limitations for a taxon of interest or prior expectations given the experimental design, for example, during ITS2 metabarcoding studies attempting to detect *Trichoderma* in plant root communities following its application as a biocontrol agent (e.g., 63). Despite their limitations, metabarcoding studies of novel or under-characterized microbial communities are important and necessary.

Most importantly, this study demonstrates how a seemingly small adjustment to data preprocessing can significantly impact the biological conclusions drawn from an analytical interpretation. Without sliding window trimming, environmental *T. septentrionalis* fungus gardens appeared to have perplexingly little microfungal diversity (Fig. 3, left). In contrast, trimmed reads revealed that environmental fungus gardens host a more diverse and abundant fungal community, of which *Trichoderma* is exceptionally prevalent (Fig. 3, right, Fig. 4). This latter result better agrees with the presence of metabolites commonly associated with *Trichoderma* in ant fungus gardens (55), other studies that have cultured many different microfungi (including *Trichoderma*) from ant fungus gardens (64–70), and the known abundance of *Trichoderma* in diverse soil and plant-associated communities such as the rhizosphere (71–75) where it often acts as a mycoparasite (76–78). *Trichoderma* is one of the most common fungi isolated from soil using culture-based techniques but is often absent or at low abundances using metabarcoding (79–81). As a result, *Trichoderma* has more often been studied in isolation using taxon-specific sequencing techniques in preference to ITS2 metabarcoding (82–84). Unexpectedly, we further discovered a strikingly similar bias against ITS2 reads from an unrelated genus, *Meyerozyma* (Fig. 5F to J), that belongs to a different subphylum (Saccharomycotina) than *Trichoderma* (Pezizomycotina). In our other research projects, we found another similar quality bias against ITS2 reads from *Clonostachys* (Fig. S7), a genus more closely related to *Trichoderma*, both in the order Hypocreales. Rolling *et al*. (54) reported a similar taxon-specific aberration in read quality for different taxa than those studied here and using ITS1 metabarcoding. Therefore, although the full distribution of such biases across all fungal taxonomy and barcodes is unknown, these data suggest they could be widespread. In conclusion, we recommend using sliding window quality trimming, appropriate quality controls, and mindful data analysis as part of best practices for metabarcoding, particularly for fungi.

## MATERIALS AND METHODS

### Data generation

Except for the cultivar fungus:*Trichoderma* DNA mock communities, all data and methods have been described elsewhere (55). The mock communities were prepared by extracting genomic DNA from pure cultures of *Trichoderma* and the *T. septentrionalis* cultivar fungus isolated from laboratory colony JKH000219, which was collected from Florida in 2016 (Florida Department of Agriculture and Consumer Services unnumbered Letter of Authorization; 55). *Trichoderma* strain JKS001884 was grown on Potato Dextrose Agar (PDA, Difco)+antibiotics (ABX, 50 mg/L penicillin and 50 mg/L streptomycin; both Fisher Scientific) at 25°C for 1 week. Cultivar fungus was isolated by collecting small tufts of hyphae from the JKH000219 fungus garden using sterile extra-fine forceps (being careful to only collect hyphae and not surrounding pieces of forage) and growing them on PDA + ABX plates at 25°C. These were checked daily for pathogen (non-cultivar) growth, in which case pathogens were cut and removed from the agar plates using sterile blades or cultivar hyphae were transferred onto new PDA + ABX plates. This continued until the cultivar fungus comprised a pure culture (~2–4 weeks).

Hyphae from these pure cultures were collected into bead-beating tubes with 250 µL of cetyltrimethylammonium bromide buffer and 0.5 g each of 0.1 mm and 1 mm sterile silica/zirconium beads for DNA extraction, and extracted DNA was quantified using a Qubit 3.0 with a dsDNA high sensitivity kit (55). Five mock communities were created using ratios of 100:0, 90:10, 50:50, 10:90, and 0:100 cultivar fungus to *Trichoderma* genomic DNA by mass. These mock communities were submitted to the Microbial Analysis, Resources, and Services facility at the University of Connecticut for ITS2 metabarcode sequencing using Illumina indexed primers fITS7 (aka ITS3, 5′-GTGARTCATCGAATCTTTG-3′, 60) and ITS4 (5′-TCCTCCGCTTATTGATATGC-3′, 85) that contained Illumina adapters and dual eight base indices (49). Samples were amplified from 30 ng of extracted DNA in triplicate 15 µL reactions using Go-Taq DNA polymerase (Promega) with the addition of 3.3 µg bovine serum albumin (New England BioLabs). To overcome inhibition from host DNA, 0.1 pmol of each primer without the adapters or indexes was added to the master mix. The ITS2 PCR reaction was incubated at 95°C for 2 minutes, then for five cycles of 30 s at 95.0°C, 60 s at 48.0°C and 60 s at 72.0°C, then for 25 cycles of 30 s at 95.0°C, 60 s at 55.0°C, and 60 s at 72.0°C, followed by final extension at 72.0°C for 10 minutes. PCR products were pooled for quantification and visualization using a QIAxcel with a DNA Fast Analysis cartridge (Qiagen). PCR products were normalized based on the concentration of DNA from 250 to 400 bp and then pooled using the epMotion 3075 liquid handling robot. The pooled PCR products were cleaned using Omega Bio-Tek Mag-Bind Beads according to the manufacturer’s protocol using a ratio of 0.8× beads to PCR product. The cleaned pool was sequenced on the MiSeq using a v2 2 × 250 base pair kit (Illumina, Inc).

### Data analysis

All ITS2 amplicon data sets were analyzed using R v3.6.3 or 4.1.0 (86). The untrimmed data set was processed following the DADA2 “ITS Pipeline Workflow (1.8)” (37, 52). The only changes were setting parameter “randomize” to “TRUE” for learning errors with the function “learnErrors,” dereplicating the forward and reverse reads using the function “derepFastq” before running the “dada” function, graphing the output of the “track” variable using R barplot to visualize read retention at each pipeline step, and using ITSx v1.1.3 (87) with default parameters to remove potential flanking 18S rRNA regions from the ASVs prior to the taxonomic classification. ASVs were removed that did not pass ITSx filtering and duplicate ITSx-treated ASVs were merged. These ASVs were then classified as described in (52) using the UNITE database v8.2 general fasta format (88). The resulting ASV table, taxon table, and sample data table were collected into a phyloseq object for further processing using phyloseq v1.26.1 (89) and tidyr v1.3.1 (90). For the trimmed data set, reads were processed exactly as above except FASTQ files were first trimmed at the 3′ end using Trimmomatic v0.39 (56) with parameters SLIDINGWINDOW:5:20. This means that each read is scanned from the 5′ end to the 3′ end using a window size of 5 bp and a quality threshold of Phred 20. Once the average quality of the five bases within the window falls below 20, the read is trimmed at the start of that window. This discards only the bases from the beginning of the failed window to the 3′ end of the read while retaining the prior higher-quality bases.

For each sample, ASVs that were <1% abundant were manually classified as “other” and their abundances were combined. *T. septentrionalis* ants predominantly cultivate fungi from the tribe *Leucocoprineae* (Agaricales: *Agaricaceae*), typically annotated as either genus *Leucocoprinus* or *Leucoagaricus* (91–93). However, the taxonomy of these fungi is complex (93–95), and, thus, ASVs were manually defined as “cultivar fungus” if they were classified as belonging to *Leucocoprinus*, *Leucoagaricus*, or “unclassified family *Agaricaceae*” These cultivar fungus ASVs were confirmed to be closely related to other fungus-growing ant cultivar fungi using NCBI blastN megablast against the nonredundant nucleotide database, nr/nt (96, 97). All code used for analysis is available at https://github.com/kek12e/ms_ITS2trimming.

## Data Availability

Unprocessed fungal ITS2 community amplicon sequencing FASTQ files are publicly available in the National Center for Biotechnology Information (NCBI) Sequence Read Archive (SRA) under BioProject PRJNA763335 (environmental fungus gardens, 98), PRJNA743045 (*Trichoderma*-infected laboratory fungus gardens, 99), and PRJNA1138067 (cultivar fungus:*Trichoderma* mock communities and *Clonostachys*, 100).

## SUPPLEMENTAL MATERIAL

Supplemental material aem.01537-24-s0001.docx Figures S1 to S7 and Table S1.

ASM does not own the copyrights to Supplemental Material that may be linked to, or accessed through, an article. The authors have granted ASM a non-exclusive, world-wide license to publish the Supplemental Material files. Please contact the corresponding author directly for reuse.
