# Supplementary material for: Untrimmed ITS2 metabarcode sequences cause artificially reduced abundances of specific fungal taxa
Source: Appl Environ Microbiol. 2024 Dec 26;91(1):e01537-24. doi: 10.1128/aem.01537-24 (PMC11784184; doi:10.1128/aem.01537-24)
Supplement: Supplemental material — Figures S1 to S7 and Table S1. [file aem.01537-24-s0001.docx]

**Supplementary Material**

[Figure S1. Untrimmed *Trichoderma* ITS2 reads are consistently filtered out or fail to merge due to unusually low read quality across the entire infection experiment. 4](#_Toc182821084)

[Figure S2. Untrimmed forward (top) and reverse (bottom) read quality plots for the mock communities replicate the bias in *Trichoderma* read quality. 5](#_Toc182821085)

[Figure S3. Sliding window trimming improves the quality of forward (top) and reverse (bottom) reads in the mock community.. 6](#_Toc182821086)

[Figure S4. Sliding window trimming rescues *Trichoderma* ITS2 reads in the infection experiment. 8](#_Toc182821087)

[Figure S5. Sliding window trimming increases the total number of reads that pass filtering and merging for all taxa in the environmental ITS2 dataset. 10](#_Toc182821088)

[Figure S6. Sliding window trimming (right) improves cultivar fungus classification in both the mock community and environmental datasets. 11](#_Toc182821089)

[Figure S7. *Clonostachys* has abnormally low read quality similar to, but distinct from, that of *Trichoderma* and *Meyerozyma*. 12](#_Toc182821090)

[Table S1. Untrimmed vs. trimmed read counts at each pipeline step for the environmental ITS2 dataset. 13](#_Toc182821091)


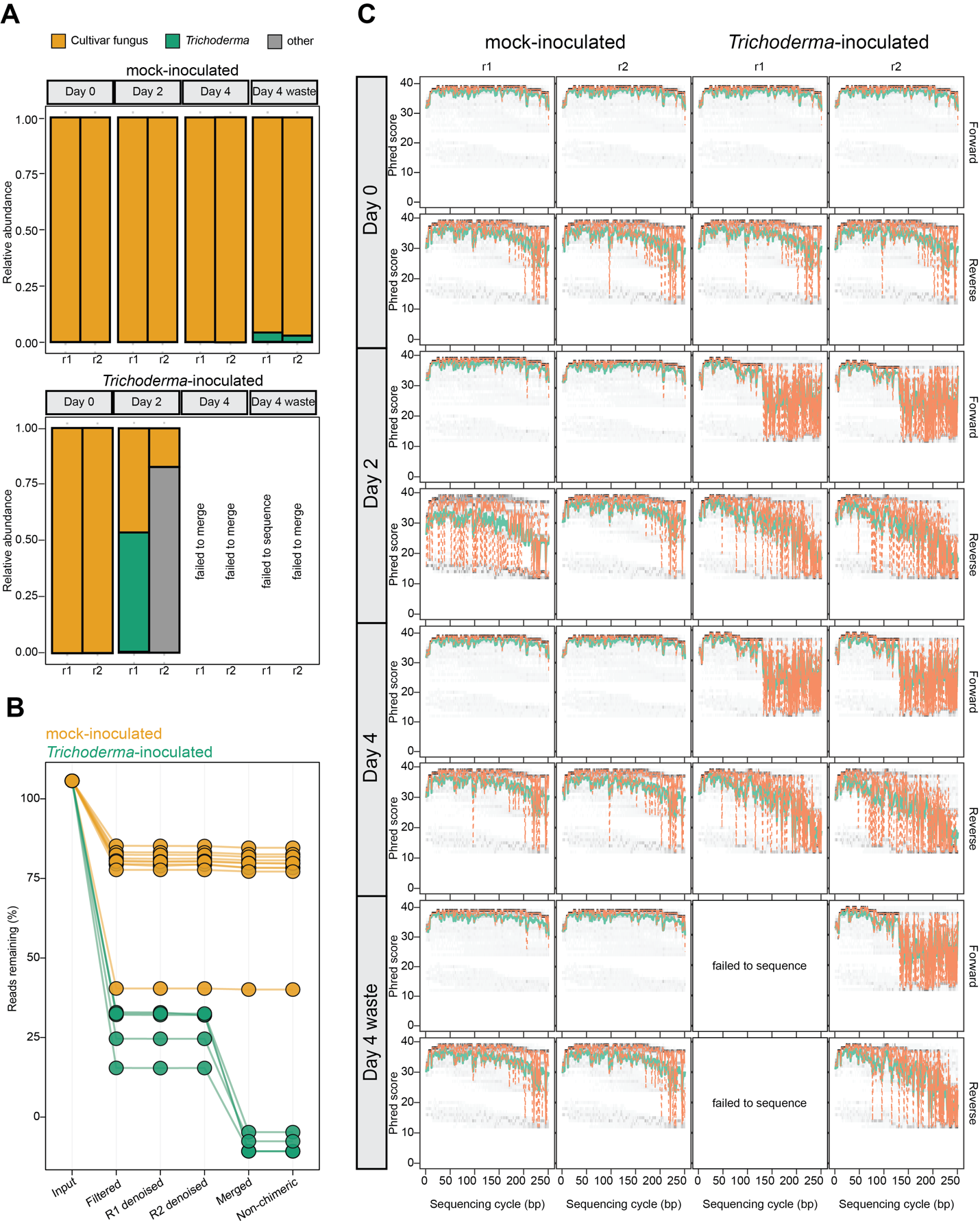


# Figure S1. Untrimmed *Trichoderma* ITS2 reads are consistently filtered out or fail to merge due to unusually low read quality across the entire infection experiment dataset. A) Relative abundance stacked bar charts using untrimmed reads for the time course *Trichoderma* infection experiment shown in Fig. 1. Days 0, 2, and 4 follow inoculation with either *Trichoderma* spores (*“Trichoderma*-inoculated”) or sterile PBS (“mock-inoculated”). Fungus garden waste (i.e., pieces removed from the fungus garden by the ants) was only collected on day 4. See Kyle *et al*. (55) for a full description of this experiment. B) Percent of untrimmed reads remaining after each sequential step of the metabarcoding pipeline for the same samples shown in (A). Forward and reverse reads are abbreviated as R1 and R2, respectively. C) Forward and reverse untrimmed read quality profiles for all samples. The mean and median quality scores for each base position are plotted as solid green and orange lines, respectively, and the 25% and 75% quality quartiles as dotted orange lines. The heatmap behind the line plots shows the basecall quality distribution for all reads in each dataset, with darker colors corresponding to more bases having that quality score.


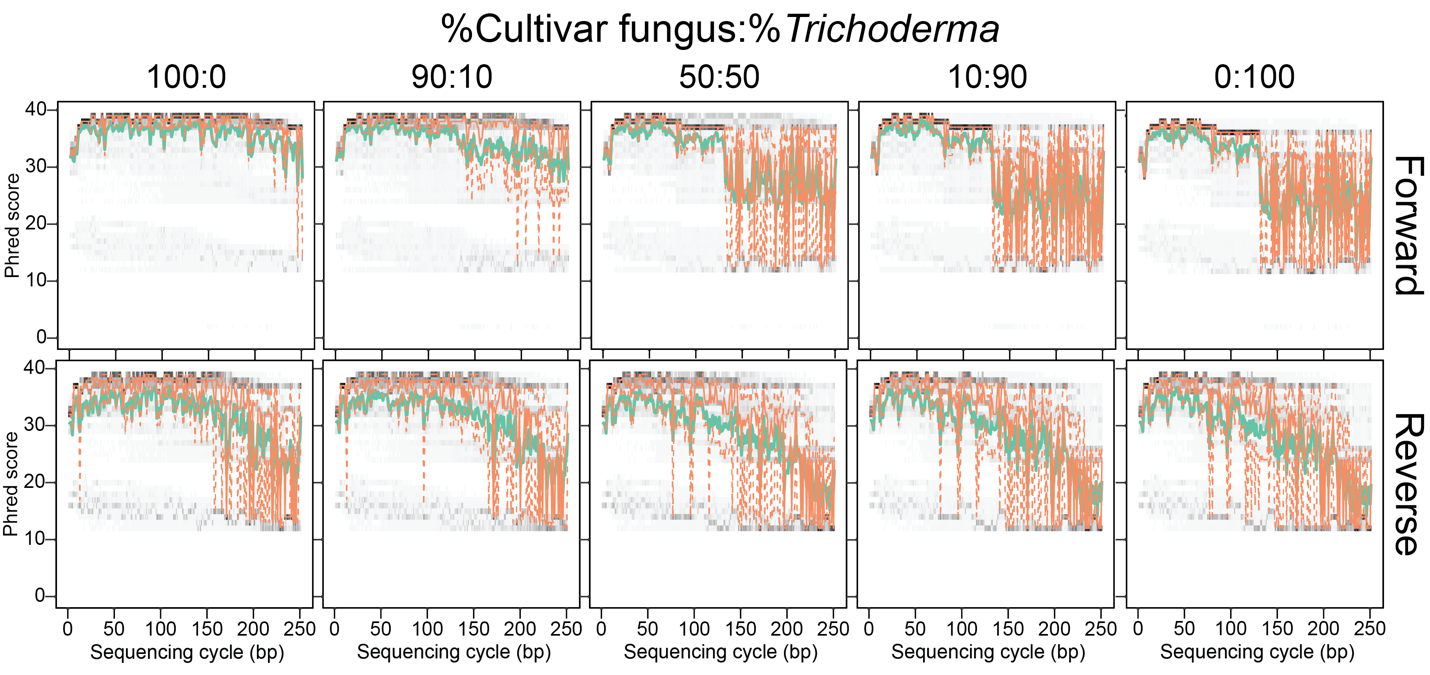


# Figure S2. Untrimmed forward (top) and reverse (bottom) read quality plots for the mock communities replicate the bias in *Trichoderma* read quality. The mean and median quality scores for each base position are plotted as solid green and orange lines, respectively, and the 25% and 75% quality quartiles as dotted orange lines. The heatmap behind the line plots shows the basecall quality distribution for all reads in each dataset, with darker colors corresponding to more bases having that quality score.


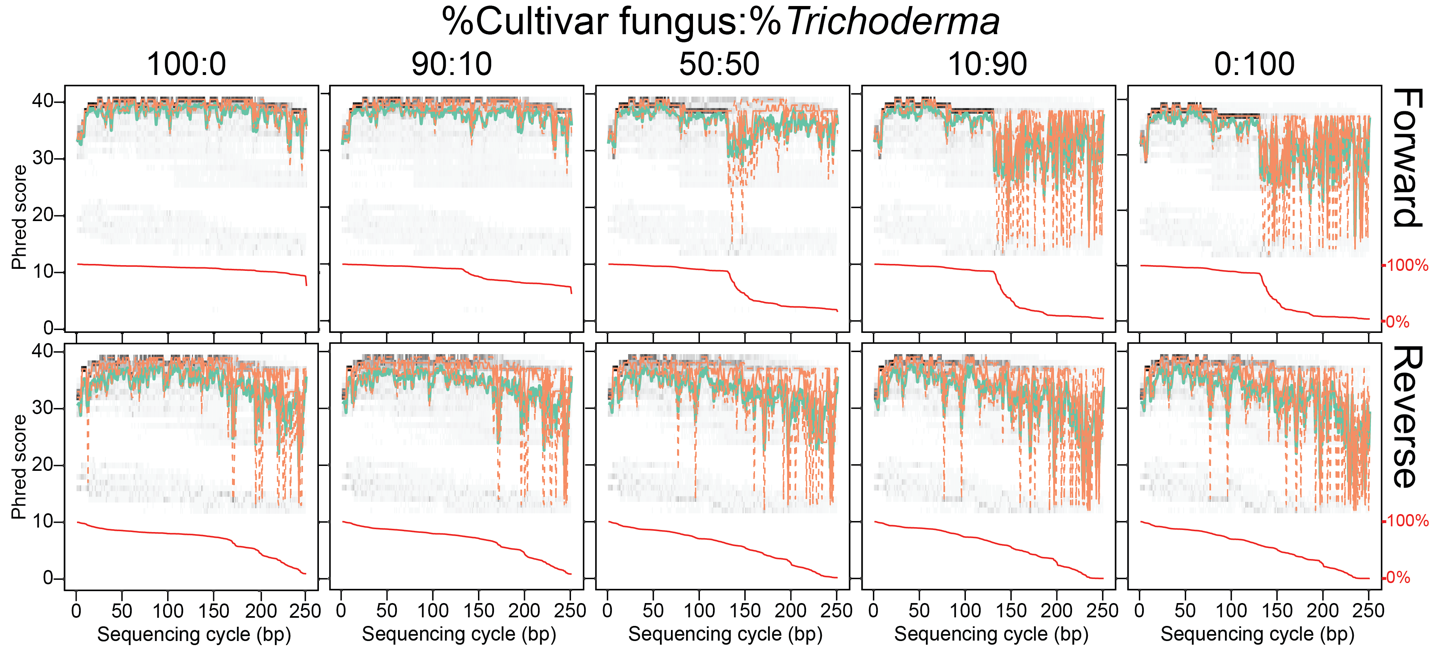


# Figure S3. Sliding window trimming improves the quality of forward (top) and reverse (bottom) reads in the mock community. The mean and median quality scores for each base position are plotted as solid green and orange lines, respectively, and the 25% and 75% quality quartiles as dotted orange lines. The heatmap behind the line plots shows the basecall quality distribution for all reads in each dataset, with darker colors corresponding to more bases having that quality score. The red line below the quality scores indicates the percentage of reads of at least that sequence length that remain after trimming.


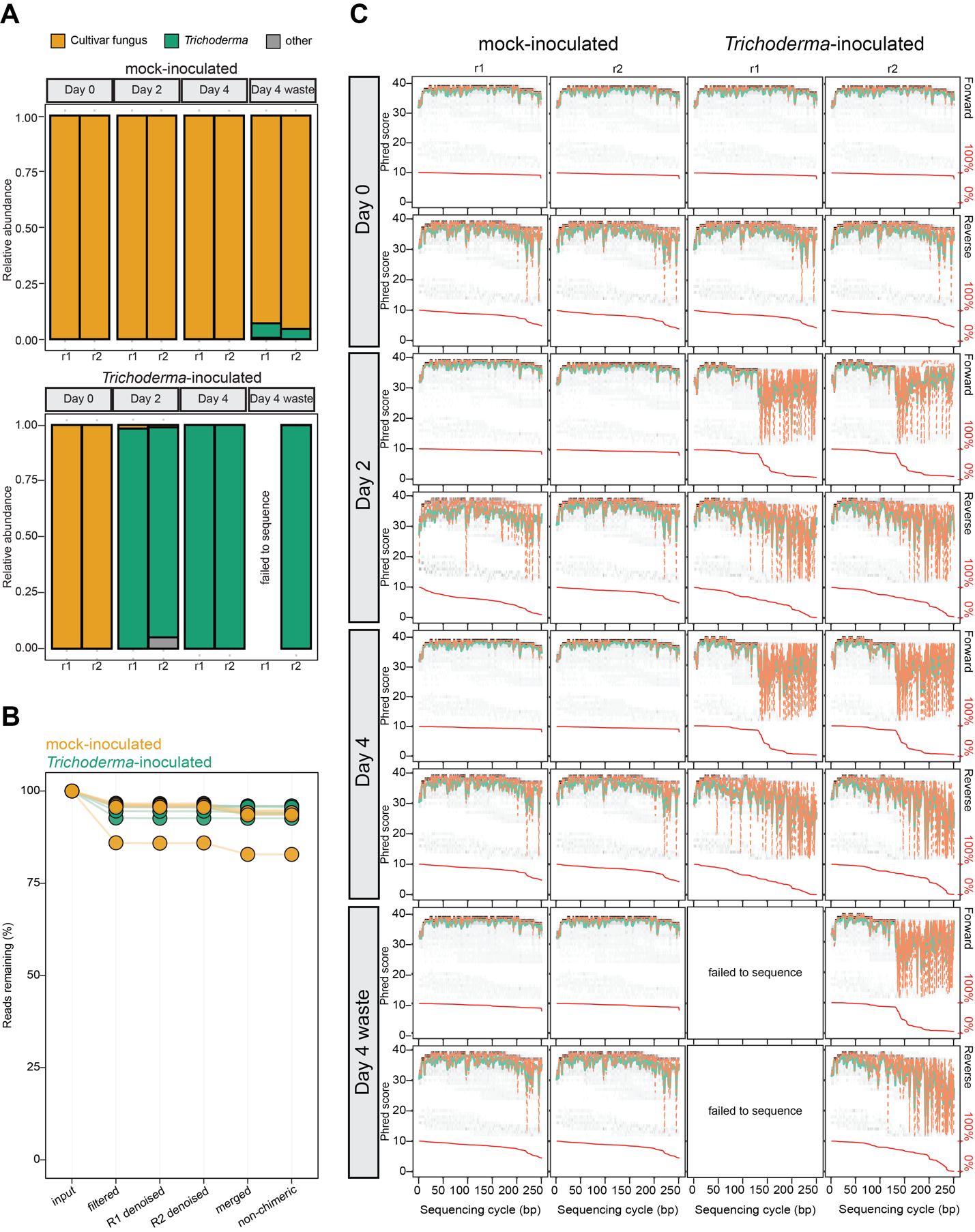


# Figure S4. Sliding window trimming rescues *Trichoderma* ITS2 reads in the infection experiment dataset. A) Relative abundance stacked bar charts using trimmed reads for the time course *Trichoderma* infection experiment shown in Fig. 1 and Suppl. Fig. S1. Days 0, 2, and 4 follow inoculation with either *Trichoderma* spores (*“Trichoderma*-inoculated”) or sterile PBS (“mock-inoculated”). Fungus garden waste (i.e., pieces removed from the fungus garden by the ants) was only collected on day 4. See Kyle *et al*. (55) for a full description of this experiment. B) Percent of trimmed reads remaining after sequential steps of the metabarcoding pipeline for the same samples shown in (A). Forward and reverse reads are abbreviated as R1 and R2, respectively. C) Forward and reverse read quality profiles for all samples. The mean and median quality scores for each base position are plotted as solid green and orange lines, respectively, and the 25% and 75% quality quartiles as dotted orange lines. The heatmap behind the line plots shows the basecall quality distribution for all reads in each dataset, with darker colors corresponding to more bases having that quality score. The red line below the quality score data shows the percentage of reads of at least that sequence length that remain after trimming.


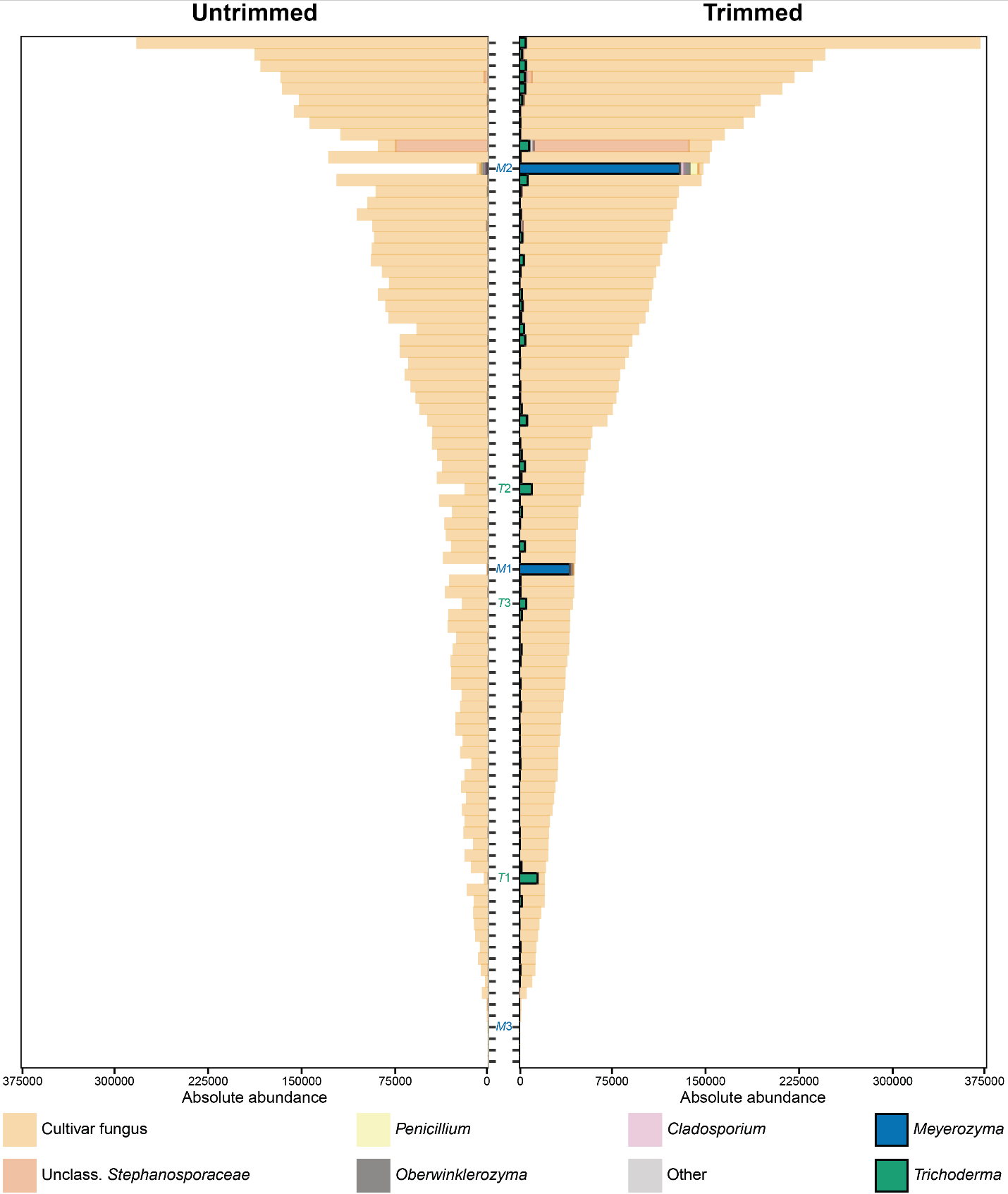


# Figure S5. Sliding window trimming increases the total number of reads that pass filtering and merging for all taxa in the environmental ITS2 dataset. Absolute abundances of environmental ITS2 reads before (left) and after (right) trimming are ordered by decreasing absolute abundance of trimmed reads. The data used are the same as in Fig. 3, and *T*1-3 and *M*1-3 are the samples containing the three highest relative abundances of *Trichoderma* and *Meyerozyma*, respectively. Suppl. Table S1 provides read counts corresponding to this figure.


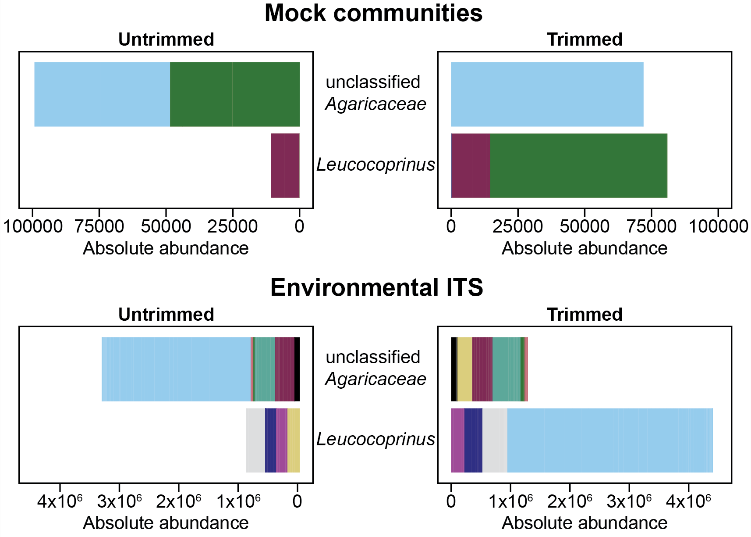


# Figure S6. Sliding window trimming (right) improves cultivar fungus classification in both the mock community and environmental datasets. Individual ASVs are represented by different colors independently for each dataset. The mock community datasets (top) contained 5 cultivar fungus ASVs both before (left) and after trimming (right). The environmental dataset (bottom) contained 74 and 63 cultivar fungus ASVs before (left) and after trimming (right), respectively. Only the top 10 cultivar fungus ASVs of the trimmed environmental dataset are shown, comprising 92.5% of all cultivar fungus reads.


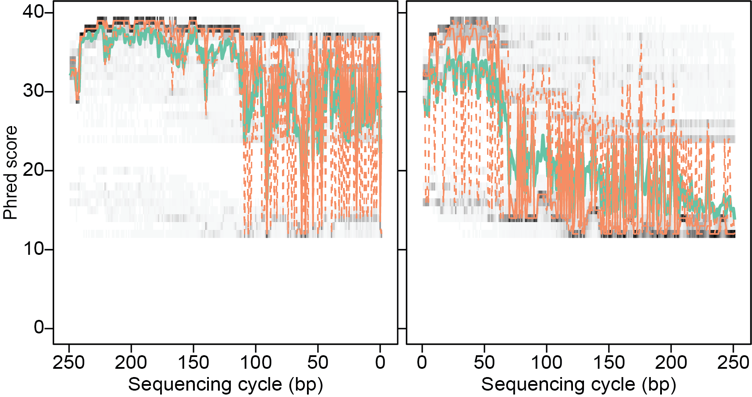


# Figure S7. *Clonostachys* has abnormally low read quality similar to, but distinct from, that of *Trichoderma* and *Meyerozyma*. ITS2 read quality for genus *Clonostachys* forward and reverse reads are shown on the left and right, respectively. The mean and median quality scores for each base position are plotted as solid green and orange lines, respectively, and the 25% and 75% quality quartiles as dotted orange lines. The heatmap behind the line plots shows the basecall quality distribution for all reads in each dataset, with darker colors corresponding to more bases having that quality score.

# Table S1. Untrimmed vs. trimmed read counts at each pipeline step for the environmental ITS2 dataset. Samples are in the same order (top to bottom) as Fig. 3. Samples *T*1-3 (green) and *M*1-3 (blue) correspond to the samples with the highest relative abudances of *Trichoderma* and *Meyerozyma*, respectively.

|  | **Untrimmed** | | | **Trimmed** | | |
| --- | --- | --- | --- | --- | --- | --- |
| **sample** | **input** | **filtered** | **merged** | **input** | **filtered** | **merged** |
| **JKA003741 (*T*1)** | 24450 | 3120 | 3029 | 21865 | 20283 | 19982 |
| **JKA003095 (*T*2)** | 73871 | 18648 | 18534 | 61946 | 54015 | 51199 |
| **JKA003520 (*T*3)** | 59653 | 26164 | 21129 | 53117 | 49037 | 42913 |
| **JKA001486** | 25424 | 11388 | 11262 | 21813 | 20301 | 20023 |
| **JKA002925** | 102736 | 36460 | 32494 | 78907 | 68558 | 48149 |
| **JKA001565** | 77813 | 37387 | 37168 | 66752 | 61741 | 53111 |
| **JKA000597** | 46977 | 15198 | 14464 | 32984 | 28116 | 22098 |
| **JKA000602** | 96529 | 50546 | 49282 | 84361 | 76960 | 71350 |
| **JKA003196** | 16443 | 5675 | 5485 | 14009 | 12775 | 12283 |
| **JKA003113** | 16524 | 6317 | 6308 | 14447 | 13133 | 13027 |
| **JKA000344** | 65028 | 2211 | 2138 | 18147 | 10566 | 10511 |
| **JKA000520** | 63470 | 37100 | 36042 | 57016 | 53175 | 44918 |
| **JKA000304** | 115238 | 71869 | 71060 | 102744 | 96243 | 90114 |
| **JKA003619** | 198161 | 88911 | 88489 | 174133 | 157854 | 155259 |
| **JKA001596** | 51340 | 28910 | 28758 | 44468 | 41124 | 40570 |
| **JKA000660** | 110882 | 31049 | 29969 | 72045 | 59380 | 50545 |
| **JKA003013** | 1126 | 668 | 651 | 979 | 901 | 856 |
| **JKA002778** | 221591 | 135480 | 133844 | 196119 | 183354 | 157880 |
| **JKA001653** | 125769 | 67331 | 60428 | 110591 | 102469 | 99638 |
| **JKA002875** | 31277 | 8504 | 8346 | 20577 | 16766 | 13155 |
| **JKA001544** | 98815 | 45242 | 44850 | 84119 | 77233 | 59413 |
| **JKA001294** | 55200 | 28275 | 25896 | 49538 | 46476 | 38811 |
| **JKA000650** | 47203 | 30399 | 29814 | 42865 | 40271 | 36551 |
| **JKA001725** | 44565 | 21121 | 15807 | 38908 | 36023 | 33789 |
| **JKA000628** | 164964 | 102000 | 100104 | 145030 | 137106 | 120821 |
| **JKA000594** | 92623 | 51875 | 49738 | 79420 | 73301 | 61030 |
| **JKA003571** | 48405 | 30592 | 30279 | 42699 | 40223 | 38551 |
| **JKA003000** | 96862 | 54884 | 54633 | 82475 | 75932 | 74333 |
| **JKA003497** | 131004 | 84104 | 82755 | 116903 | 109486 | 103514 |
| **JKA000610** | 57452 | 35970 | 35265 | 51281 | 48116 | 44423 |
| **JKA000558** | 46122 | 11796 | 11648 | 31505 | 24790 | 22905 |
| **JKA002913** | 90100 | 34085 | 33293 | 68343 | 58555 | 46296 |
| **JKA001174** | 82243 | 25768 | 25480 | 59548 | 51796 | 35153 |
| **JKA000348** | 267716 | 167290 | 165834 | 237992 | 222640 | 210172 |
| **JKA003025** | 296427 | 188085 | 186014 | 260363 | 245992 | 238379 |
| **JKA000641** | 154313 | 93347 | 92038 | 132608 | 123868 | 111079 |
| **JKA003083** | 146021 | 93979 | 93448 | 130388 | 122663 | 120168 |
| **JKA001204** | 46708 | 22483 | 22176 | 41055 | 37650 | 26324 |
| **JKA003144** | 334387 | 175302 | 168707 | 301904 | 284014 | 223229 |
| **JKA000631** | 31014 | 11679 | 11384 | 22165 | 19315 | 16548 |
| **JKA001085** | 47129 | 22804 | 22327 | 41397 | 38150 | 34347 |
| **JKA001704** | 38599 | 18685 | 18584 | 33491 | 30445 | 30182 |
| **JKA003878** | 50201 | 31721 | 26207 | 43797 | 41102 | 32599 |
| **JKA000906** | 59909 | 21109 | 20922 | 43262 | 36652 | 35094 |
| **JKA001150** | 84801 | 41330 | 40604 | 73561 | 68694 | 53810 |
| **JKA003607** | 129200 | 80838 | 80112 | 113662 | 105653 | 100607 |
| **JKA000827** | 1747 | 838 | 799 | 1541 | 1358 | 1037 |
| **JKA002832** | 78692 | 46484 | 46048 | 67615 | 62731 | 58083 |
| **JKA002896** | 30759 | 18996 | 18518 | 27009 | 25169 | 21671 |
| **JKA001620** | 486532 | 300018 | 299499 | 427782 | 397096 | 394447 |
| **JKA002747** | 67671 | 40414 | 39496 | 59044 | 54778 | 47881 |
| **JKA003662** | 290590 | 164739 | 163374 | 248356 | 228428 | 204812 |
| **JKA000263** | 158257 | 108463 | 106548 | 144112 | 134756 | 123218 |
| **JKA002341** | 63323 | 36731 | 35815 | 54048 | 50260 | 43658 |
| **JKA001100** | 132152 | 73493 | 70813 | 117827 | 111211 | 88900 |
| **JKA003815** | 62701 | 39871 | 39394 | 55725 | 52076 | 48897 |
| **JKA003645** | 73996 | 25975 | 25551 | 52153 | 44847 | 39604 |
| **JKA003836** | 145191 | 86737 | 85564 | 126135 | 116974 | 109276 |
| **JKA003591** | 103177 | 63656 | 63594 | 90789 | 84902 | 84477 |
| **JKA000222** | 319503 | 190385 | 188426 | 282102 | 257923 | 244784 |
| **JKA002735** | 165319 | 93291 | 91801 | 144557 | 132499 | 129959 |
| **JKA000690** | 42030 | 26089 | 25872 | 37184 | 34196 | 33012 |
| **JKA001126** | 142455 | 74814 | 72581 | 125969 | 118106 | 94084 |
| **JKA001308** | 32935 | 21260 | 21226 | 29479 | 27598 | 27046 |
| **JKA003712** | 201984 | 131906 | 129516 | 176517 | 166106 | 152692 |
| **JKA002762** | 56535 | 17749 | 17420 | 39421 | 32897 | 27321 |
| **JKA000614** | 19960 | 10159 | 10075 | 16894 | 14996 | 14436 |
| **JKA003463** | 106880 | 71279 | 70835 | 95744 | 90266 | 87293 |
| **JKA001165** | 36520 | 13482 | 13040 | 27798 | 23660 | 18455 |
| **JKA003827** | 29189 | 19121 | 18786 | 26143 | 24242 | 22789 |
| **JKA001184** | 34136 | 21955 | 21792 | 30837 | 28787 | 28352 |
| **JKA003485** | 202996 | 108034 | 105235 | 175188 | 158069 | 133494 |
| **JKA000547** | 232145 | 145709 | 144161 | 203573 | 189446 | 179213 |
| **JKA003450** | 16673 | 4919 | 4753 | 12041 | 5660 | 5457 |
| **JKA001123** | 158260 | 97685 | 97001 | 138892 | 130089 | 125802 |
| **JKA003581** | 225481 | 119675 | 118750 | 191399 | 171234 | 164235 |
| **JKA003766** | 46964 | 29443 | 29114 | 41434 | 38134 | 36566 |
| **JKA003852** | 61219 | 33862 | 33584 | 51040 | 46586 | 44786 |
| **JKA001227** | 141541 | 82863 | 82445 | 123061 | 112937 | 109791 |
| **JKA003473** | 228563 | 157975 | 156794 | 207247 | 195259 | 188813 |
| **JKA000625** | 99760 | 68243 | 67675 | 90096 | 85068 | 80985 |
| **JKA003156** | 155 | 50 | 40 | 120 | 104 | 68 |
| **JKA003440** | 121 | 56 | 56 | 93 | 60 | 58 |
| **JKA000355** | 154149 | 94953 | 93880 | 137875 | 127403 | 113970 |
| **JKA003559 (*M*1)** | 72987 | 23086 | 873 | 54766 | 47639 | 44112 |
| **JKA003634 (*M*2)** | 283 | 147 | 26 | 235 | 197 | 191 |
| **JKA003790 (*M*3)** | 202255 | 85671 | 10583 | 177789 | 160866 | 150132 |
| **JKA003864** | 37851 | 21261 | 21140 | 32542 | 29478 | 28366 |
| **JKA000507** | 98294 | 50565 | 49342 | 83725 | 75777 | 62797 |
| **JKA001243** | 305 | 60 | 40 | 191 | 141 | 0 |
